# *Acinetobacter baumannii* Infection in Prior ICU Bed Occupants Is an Independent Risk Factor for Subsequent Cases of Ventilator-Associated Pneumonia

**DOI:** 10.1155/2014/193516

**Published:** 2014-07-02

**Authors:** Eirini Tsakiridou, Demosthenes Makris, Zoe Daniil, Efstratios Manoulakas, Vasiliki Chatzipantazi, Odysseas Vlachos, Grigorios Xidopoulos, Olympia Charalampidou, Epaminondas Zakynthinos

**Affiliations:** ^1^Department of Critical Care Medicine, University Hospital of Larisa and School of Medicine, University of Thessaly, Biopolis, 41000 Larisa, Greece; ^2^Department of Respiratory Medicine, University Hospital of Larisa and School of Medicine, University of Thessaly, Biopolis, 41000 Larisa, Greece; ^3^Department of Critical Care Medicine, General Hospital of Serres, 62100 Serres, Greece

## Abstract

*Objective*. We aimed to evaluate risk factors for ventilator-associated pneumonia (VAP) due to *Acinetobacter baumannii* (AbVAP) in critically ill patients. *Methods*. This was a prospective observational study conducted in an intensive care unit (ICU) of a district hospital (6 beds). Consecutive patients were eligible for enrolment if they required mechanical ventilation for >48 hours and hospitalization for >72 hours. Clinical, microbiological, and laboratory parameters were assessed as risk factors for AbVAP by univariate and multivariate analysis. *Results*. 193 patients were included in the study. Overall, VAP incidence was 23.8% and AbVAP, 11.4%. Previous hospitalization of another patient with *Acinetobacter baumannii* infection was the only independent risk factor for AbVAP (OR (95% CI) 12.016 (2.282–19.521) *P* < 0.001). ICU stay (25 ± 17 versus 12 ± 9  *P* < 0.001), the incidence of other infections (OR (95% CI) 9.485 (1.640–10.466) *P* = 0.002) (urinary tract infection, catheter related infection, and bacteremia), or sepsis (OR (95% CI) 10.400 (3.749–10.466) *P* < 0.001) were significantly increased in patients with AbVAP compared to patients without VAP; no difference was found with respect to ICU mortality. *Conclusion*. ICU admission or the hospitalization of patients infected by *Acinetobacter baumannii* increases the risk of AbVAP by subsequent patients.

## 1. Introduction

Ventilator-associated pneumonia (VAP) may cause considerable morbidity [1, 2]. The risk of VAP is related to several host and treatment factors [3].

In recent years,* Acinetobacter baumannii* has become one of the predominant organisms responsible for VAP. Despite advances in prevention, outbreaks especially with multidrug-resistant strains of this organism have emerged as a major problem in the intensive care unit (ICU) [4]. Previous studies reported several risk factors for* Acinetobacter baumannii* VAP (AbVAP), such as elevated APACHE II score, previous antibiotic exposure to particular antibiotic agents, Glasgow coma score ≤ 9, head injury, ARDS, neurosurgery and large volume pulmonary aspiration [5–8]. In Greece, despite the fact that* Acinetobacter baumannii *infection is a major clinical problem, data regarding risk factors for AbVAP are limited [9, 10].

In the present prospective study, we therefore aimed to investigate potential risk factors for AbVAP and to study clinical outcomes of ICU patients with AbVAP in comparison with critically ill patients who did not present VAP or presented VAP caused by other pathogens.

## 2. Methods

This prospective observational study was conducted in a general ICU of a district hospital in Greece. During a 2-year period (2010−2012), all consecutive patients who were admitted to the ICU were screened for eligibility and entered the study if they met the following two criteria: (a) intubation for more than 48 hours and (b) hospitalization for more than 72 hours. The study was approved by the University of Thessaly (UT 08-02-10/568) and the International Centre of Services of Health Ministry (UT 01-06-10/2124).

## 3. Study Groups

Patients were classified as patients with AbVAP, patients with VAP due to other pathogens, and patients without VAP.

## 4. Outcomes

We primarily assessed risk factors for AbVAP. Secondarily, we compared ICU mortality, ICU stay, and the incidence of other infections between patients with AbVAP and patients in the other two study groups.

## 5. Clinical Evaluation

Baseline demographic and clinical data including age, sex, BMI, APACHE II score, and cause of admission were recorded. Data from patients' medical history including previous antibiotic exposure, previous hospitalizations, corticosteroids exposure, or any* Acinetobacter baumannii* infection on admission were also recorded. The hospitalization in the ICU of another patient with documented* Acinetobacter baumannii* infection, during the 10 days preceding the VAP diagnosis or at any time for patients without VAP, and medical interventions associated previously with VAP, such as blood transfusion, corticosteroid treatment, and enteral or parenteral feeding, were recorded daily; these interventions required being present at least 48 hours before VAP diagnosis. All patients were evaluated daily until hospital discharge, for VAP or other types of infection and clinical pulmonary infection score (CPIS) was recorded [11]. The monthly bed occupancy rate was also calculated.

## 6. Sample Collection

Blood, urine, and tracheal aspirates with a sterile sputum trap for semiquantitative cultures were collected routinely at ICU admission, but also during the hospital stay whenever there were clinical and laboratory signs of infection [12].

## 7. Definitions 

VAP was defined as a new persistent chest-radiographic infiltrate in conjunction with one of the following: a positive blood or pleural fluid culture or two of the following: fever (temperature > 38.3°C), leukocytosis (leukocyte count > 10^4^/mm^3^), and purulent tracheal aspirate [13]. In addition, a positive tracheal aspirate culture was required to confirm the diagnosis of VAP [11]. Blood stream infections, urinary tract infections, and intravenous catheter related infections were defined according to CDC/NHSN definition of healthcare-associated infections [14]. Sepsis was defined as previously suggested by international consensus guidelines [15]. Multidrug-resistant (MDR) bacteria included methicillin resistant* Staphylococcus aureus*, ceftazidime or imipenem resistant* Pseudomonas aeruginosa*, colistin sensitive* Acinetobacter baumannii* and* Stenotrophomonas maltophilia,* and Gram-negative bacilli producing extended spectrum beta lactamase [2]. The overall monthly bed occupancy rate was calculated by the formula cumulative inpatient days × 100/number of beds × days of month.

## 8. Statistics 

Data are presented as frequency (%) for qualitative parameters or mean ± SD for quantitative variables. Comparisons between groups were performed by using *t*-test/Mann-Whitney *U* tests or chi-square/Fishers exact test as appropriate. Univariate analysis was performed to determine variables potentially associated with AbVAP, such as demographics, comorbidities, corticosteroids and blood transfusion in ICU, previous antibiotic and corticosteroid exposure, previous surgery, previous infection, enteral or parenteral feeding, and previous ICU hospitalization of another patient with* Acinetobacter baumannii* infection during the 10 days preceding the VAP diagnosis or at any time for patients without VAP. Variables which were significant at the 0.05 level were then included in multivariate logistic regression analysis. Model's calibration was tested using the Hosmer-Lemeshow goodness-of-fit statistic. A high *P* value (0.05) would indicate a good fit for the model. The overall accuracy (discrimination) of the model has been evaluated too. Data were analyzed by using SPSS version 17.

## 9. Results

**Figure 1:**
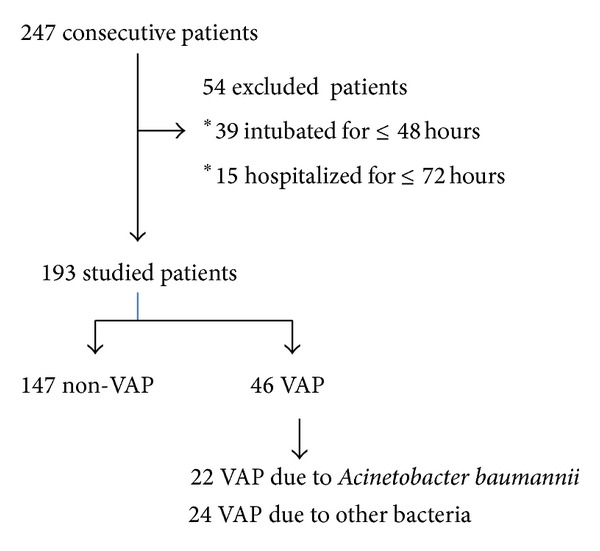
Flow chart of the study.

One hundred ninety-three patients out of 247 admissions were eligible and entered the study; fifty-four patients were excluded because they were intubated for < 48 hours or hospitalized for < 72 hours (Figure 1). There were 46 patients with VAP; 22 of them (48%) with AbVAP and 24 (52%) with VAP due to other pathogens (Table 1), which were MDR in 42% of cases (OR (95% CI) 14; (2.649–73.980) *P* < 0.001). There were no statistical differences between patients with AbVAP and patients with VAP due to other pathogens in terms of cause of admission or with respect to clinical characteristics. Similarly, no significant differences were found between patients with AbVAP and patients without VAP (Table 2).

## 10. VAP due to* Acinetobacter baumannii*

**Figure 2:**
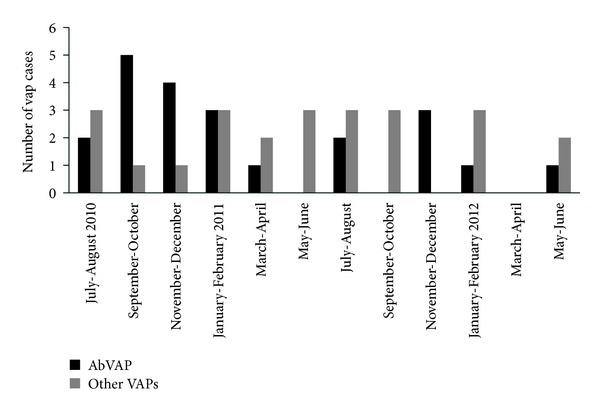
Distribution of VAP cases per two-month period.

**Figure 3:**
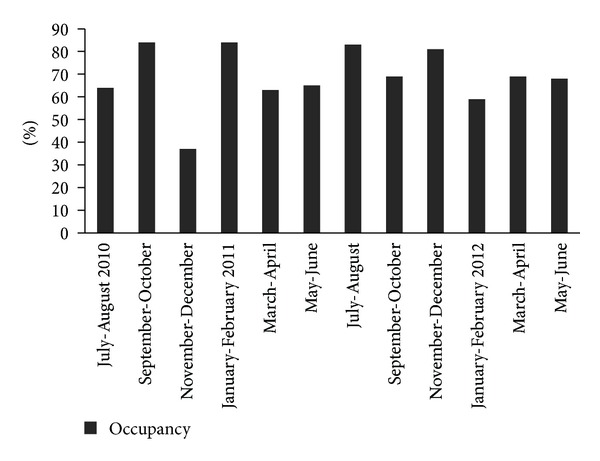
Occupancy of the ICU per two-month period.

In the majority of AbVAP cases, the cause of admission was respiratory failure (41%) or neurologic disorders (18%). The mean time for AbVAP diagnosis was 12 ± 11 days (Table 3). AbVAP compared to VAP due to other pathogens was more prevalent during the autumn and winter (*P* = 0.064) and more specifically during November and December (*P* = 0.013) (Figure 2). There was no significant difference between the occupancy rate during this period (mean rate of 59%) in the ICU and the rates observed during other months of the two-year study period (which ranged between 66% and 77%, *P* = 0.278) (Figure 3).

Enteral feeding in ICU (OR (95% CI) 2.927; (1.026–8.351) *P* = 0.038), blood transfusion in ICU (OR (95% CI) 2.591; (1.014–6.617) *P* = 0.041), and previous hospitalization of another patient with* Acinetobacter baumannii* infection (OR (95% CI) 7.013; (2.442–20.140) *P* < 0.001) were significantly associated with the presence of AbVAP (Table 3). Multivariate analysis showed that previous hospitalization of another patient with* Acinetobacter baumannii* infection (OR (95% CI) 6.674; (2.282–19.521) *P* < 0.001) was the only independent risk factor for AbVAP (Table 4). When characteristics of patients with AbVAP were compared to those of patients with VAP due to other pathogens, no independent risk factor for AbVAP was identified.

## 11. VAP Outcome due to* Acinetobacter baumannii*

Patients with AbVAP presented significantly increased incidence of other types of infection (urinary tract infection, intravenous catheter related infection, and bacteremia) (OR (95% CI) 4.143; (1.640–10.466) *P* = 0.002), due to MDR bacteria (OR (95% CI) 9.485; (3.269–25.517) *P* < 0.001), sepsis (OR (95% CI) 10.400; (3.749–28.853) *P* < 0.001), and prolonged ICU stay (25 ± 17 days versus 12 ± 9, *P* < 0.001) compared to patients without VAP (Table 5). Patients with VAP due to other MDR pathogens had significantly prolonged ICU stay compared to patients with AbVAP (39 ± 24 days versus 25 ± 17, *P* = 0.058).

ICU mortality was not significantly increased in patients with AbVAP compared to patients without VAP. Urinary tract infection (OR (95% CI) 1.875; (1.168–3.01) *P* = 0.029), previous antibiotic exposure (OR (95% CI) 16.5; (1.487–183.07) *P* = 0.01), older age (71 ± 11 years versus 51 ± 12, *P* = 0.001), and Apache II score (27 ± 5 versus 20 ± 8, *P* = 0.015) were risk factors for ICU mortality in patients with AbVAP. However, multivariate analysis did not identify any independent risk factor for death in patients with AbVAP. In addition, sepsis was the only independent risk factor for death in patients without VAP (OR (95% CI) 7.242; (2.016–26.019) *P* = 0.002). No independent risk factor for ICU mortality in patients with VAP due to other pathogens was found.

## 12. Discussion 

The main finding of this study is the association between the hospitalization of patients with* Acinetobacter baumannii *infection and the increased risk of AbVAP by subsequent patients. In addition, the present study showed that patients with AbVAP presented increased length of ICU stay, increased incidence of other ICU infections, and sepsis, compared to patients without VAP, but similar clinical outcome measures to those of patients with VAP by other bacteria.

In the present study, we evaluated several risk factors for increased frequency of AbVAP. AbVAP was found to be associated with the previous hospitalization of another patient with* Acinetobacter baumannii* infection. Prior room occupant with* Acinetobacter baumannii* has been also previously suggested as independent risk factor for ICU-aquired* Acinetobacter baumannii* from only one existing study [16]. This finding indicates that hospital personnel, admitted patients, and medical equipment are important reservoirs of the organism [17]. The fact that medical equipment and more specifically ventilators can be contaminated by* Acinetobacter baumannii *has been reported in another recent study [18]. These data underline the necessity of strict microbiological surveillance and infection control strategies for the primary prevention of the transmission of this organism in the ICU. These strategies could include rigorous cleaning and disinfecting of hands and ICU environment and implementation of an antibiotic control and therapeutic strategy according to specific guidelines, adapted to the local settings [19, 20].

Previous studies have also reported that the risk of AbVAP in intubated patients with nosocomial pneumonia was higher in patients with head trauma, neurosurgery, ARDS, large volume aspiration, and prior antibiotic use [5–8]. In the present investigation, there were not many neurosurgical patients and the number of ARDS patients was also small. Hence, we found no association between the above-mentioned factors and AbVAP. Enteral feeding was indeed associated with AbVAP in our study, but the association was not significant in multivariate analysis. However, in the present study, we found that AbVAP was more frequent in certain time periods. AbVAP incidence was increased during the humid winter months (Figure 2). This may be different to what one might have expected because previous studies reported that the incidence is higher during humid warmer months [21, 22]. Nevertheless, humidity is higher in winter months, especially during November and December, compared to other seasons in Greece; the relative humidity of the study region shows a progressive decrease during the first semester of the year, ranging between 74.4% and 53.1%, and a progressive increase during the second semester of the year, reaching a maximum level of 78.4% during November and December [23]. Therefore, seasonality in the occurrence of* Acinetobacter baumannii* infection in relation to the humidity of the geographic region could be possible.

An increased prevalence of multidrug-resistant pathogens or a higher level of occupancy/complexity with increased workload for staff during specific periods could also affect AbVAP frequency [24, 25]. However, bed occupancy rates in our department—which is an indirect index of workload—was not associated with AbVAP frequency in our study. On the other hand, our data regarding colonization rates during the whole 2-year study are limited and a possible association cannot be ascertained. A longitudinal study, which could answer adequately this question, is underway.

It should be also pointed out that VAP depends also on host-related immune factors and on the grade of virulence of each pathogen. Unfortunately, little is known about the pathogenesis and the host-pathogen interactions of* Acinetobacter baumannii* [26, 27] and our study has not depicted any risk factor that could provide further insight in these interactions.

In the present investigation, patients with AbVAP presented significantly increased frequency of other types of MDR infections and sepsis and had prolonged ICU stay compared to patients without VAP, while no differences were found with respect to the ICU mortality. Previous studies have pointed out that* Acinetobacter baumannii* infection may increase the length of ICU stay, but the relationship between* Acinetobacter baumannii* infection frequency and ICU mortality is not constant, since in many studies mortality was not significantly increased in patients with* Acinetobacter baumannii* infection compared to controls [28].

In this study, no significant differences were found in terms of length of ICU stay, mortality, other types of infection, or sepsis frequency between patients with AbVAP and patients with VAP due to other microbial agents. This is in agreement with previous studies that reported similar prognosis between patients with AbVAP and patients with VAP due to other microorganisms [8, 29]. Hence, our data do not support that VAP due to* Acinetobacter baumannii* carries a worse prognosis than other causes of VAP. Notably, patients with VAP due to other MDR bacteria presented significantly increased length of stay in the ICU compared to patients with AbVAP in our study. These findings might indicate that there are several variables involved in the clinical course of critically ill patients, such as appropriate empirical treatment and the interaction between host defense mechanisms and the pathogen. In our study, the rates of initial appropriate empirical treatment were different between AbVAP and VAP due to other MDR bacteria (68% versus 30%; *P* = 0.044). On the other hand, MDR bacteria, such as* Pseudomonas aeruginosa* or* Klebsiella* species, are more virulent or inherently difficult to be eradicated even with effective therapy, especially in patients with impaired host defenses or due to treatment differences [30, 31].

We acknowledge that there are some points that have to be taken into consideration when interpreting the results of the present study. First, we did not assess all potential risk factors that have been previously reported to be associated with VAP due to certain agents, such as colonization rates—as it was pointed out previously—or the type of previous antibiotic exposure or risk factors that could have an impact on the outcome of VAP. Thus, the possibility of missing other possible risk factors for increased VAP frequency due to* Acinetobacter baumannii* or for increased mortality cannot be excluded. Second, this is one center study, which might represent the microbiological flora of the ICU of a general district hospital, and one should be cautious in generalizing our results.

In conclusion, our study suggests that the hospitalization of patients with* Acinetobacter baumannii* infection in the ICU may increase the risk of VAP due to this bacterium by subsequent patients. Moreover, we found that AbVAP incidence might be increased during the months, when humidity is increased. AbVAP patients presented increased length of ICU stay and increased incidence of other ICU infections and sepsis compared to patients who did not present VAP. In this respect, more efforts should be addressed to infection control measures in order to prevent the transmission of this organism between ICU patients.

## Figures and Tables

**Table 1:** Microbiology of VAP.

	VAP *n* = 46
*Pseudomonas aeruginosa *	5 (11%)
*Klebsiella spp. *	6 (13%)
*Enterobacter spp. *	3 (6.5%)
*Proteus spp. *	3 (6.5%)
*Acinetobacter baumannii *	22 (48%)
*Stenotrophomonas maltophilia *	1 (2%)
CNS	3 (7%)
MSSA	1 (2%)
*Enterococcus faecium *	2 (4%)

Data are presented as *n* (%).

CNS: coagulase negative *Staphylococci* and MSSA: methicillin sensitive *Staphylococcus aureus*.

∗The results are referred to monomicrobial infections.

**Table 2:** Baseline characteristics of the study patients.

	Non-VAP *n* = 147	AbVAP *n* = 22	VAP due to other pathogens *n* = 24
Sex (male)	86 (59%)	12 (55%)	16 (67%)
Age (years)	66 ± 17	65 ± 15	66 ± 10
APACHE II score	24 ± 8	25 ± 7	23 ± 5
BMI	29 ± 8	29 ± 4	28 ± 5
*Comorbidities *			
Hypertension	83 (57%)	10 (46%)	9 (38%)
Hyperlipidemia	35 (24%)	3 (14%)	6 (25%)
Cardiac disease	76 (52%)	7 (31.8%)	7 (29%)
Stroke	18 (12%)	3 (14%)	2 (8%)
Chronic kidney disease	13 (9%)	1 (5%)	3 (13%)
Cancer	11 (8%)	1 (5%)	2 (8%)
COPD	31 (21%)	4 (18%)	2 (8.3%)
Others	41 (28%)	5 (23%)	0%
*Cause of admission *			
Cardiac disorders	29 (20%)	1 (5%)	4 (17%)
Neurologic disorders	23 (16%)	4 (18%)	6 (25%)
Respiratory failure	57 (39%)	9 (41%)	7 (29%)
Sepsis	4 (3%)	2 (9%)	2 (8%)
Trauma	14 (10%)	2 (9%)	3 (13%)
Surgical	19 (13%)	2 (9%)	2 (8%)
ARDS	10 (7%)	0%	3 (13%)
Head trauma	4 (3%)	2 (9%)	0%
Others	5 (4%)	2 (9%)	0%

Data are presented as *n* (%) or mean (±SD). Differences between the analyzed groups of the study were evaluated by *t*-test or *x* ^2^ test as appropriate.

VAP: ventilator-associated pneumonia, AbVAP: VAP due to *Acinetobacter baumannii*, BMI: body mass index, and COPD: chronic obstructive pulmonary disease.

No significant differences were observed between the 3 groups of the study.

**Table 3:** Risk factors for VAP due to *Acinetobacter baumannii*.

	Non-VAP *n* = 147	AbVAP *n* = 22	VAP due to other pathogens *n* = 24
Enteral feeding	79 (54%)∗	17 (77%)	19 (79%)
Parenteral feeding	135 (92%)	20 (91%)	21 (88%)
Blood transfusion in ICU	31 (21%)∗	9 (41%)	12 (50%)
Corticosteroids in ICU	42 (29%)	7 (32%)	8 (32%)
BSI in ICU	26 (18%)	7 (32%)	6 (25%)
Infection on admission	53 (36%)	11 (50%)	10 (42%)
Blood transfusion on admission	8 (5%)	1 (5%)	0%
Previous surgery	19 (13%)	2 (9%)	2 (8%)
Preceding intake of antibiotics	57 (39%)	12 (55%)	9 (38%)
Preceding corticosteroid treatment	7 (5%)	0%	1 (4%)
Previous hospitalization	60 (41%)	10 (46%)	9 (38%)
Previous patient with Ab infection	48 (33%)∗	17 (77%)	14 (58%)
ICU stay (prior VAP)	12 ± 9	12 ± 11	15 ± 10

Data are presented as *n* (%) or mean (±SD).

VAP: ventilator-associated pneumonia, ICU: intensive care unit, BSI: bloodstream infections, and Ab: *Acinetobacter baumannii*.

Previous patients with Ab infection were considered those who were hospitalized in ICU during 10 days preceding the VAP diagnosis or at any time for patients without VAP.

__*P value between AbVAP and non-VAP patients.

No significant differences were observed between AbVAP patients and patients with VAP by other agents.

**Table 4:** Multivariate analysis of risk factors for VAP due to *Acinetobacter baumannii*.

	OR	95% CI	Wald statistic	*P*
Enteral feeding	2.759	0.919–8.281	3.275	0.07
Another patient with Ab infection	6.674	2.282–19.521	12.016	0.001
Blood transfusion in ICU	2.106	0.766–5.792	2.083	0.149

The Hosmer-Lemeshow statistic (calibration) was not significant: chi-square = 2.466, df = 7, *P* = 0.782. Overall accuracy (discrimination) was 87%.

BSI: bloodstream infections, ICU: intensive care unit, and Ab: *Acinetobacter baumannii*.

**Table 5:** Clinical outcome measures in patients with VAP due to *Acinetobacter baumannii*.

	Non-VAP *n* = 147	AbVAP *n* = 22	VAP due to other pathogens *n* = 24
Other infections	38 (26%)∗	13 (59%)	17 (71%)
Urinary tract infection	12 (8%)∗	7 (32%)	6 (25%)
Intravenous catheter infection	15 (10%)∗	6 (27%)	9 (38%)
Bacteremia in ICU	28 (19%)∗	13 (59%)	14 (58%)
Other infections due to MDR	10 (7%)∗	9 (41%)	7 (29%)
Sepsis	30 (20%)∗	16 (73%)	19 (79%)
ICU stay (days)	12 ± 9∗	25 ± 17	34 ± 23
ICU mortality	87 (59%)	15 (68%)	15 (62%)

Data are presented as *n* (%) or mean (±SD).

VAP: ventilator-associated pneumonia, ICU: intensive care unit, and MDR: multidrug-resistant bacteria.

Other infections included bacteremia, urinary tract infection, and infection of intravenous catheter.

__*P value between AbVAP and non-VAP patients.
